# Gelatin/Na_2_Ti_3_O_7_ Nanocomposite Scaffolds: Mechanical Properties and Characterization for Tissue Engineering Applications

**DOI:** 10.3390/polym15102322

**Published:** 2023-05-16

**Authors:** Rittichai Sangkatip, Kaona Jongwuttanaruk, Wipoo Sriseubsai

**Affiliations:** 1 Department of Industrial Engineering, School of Engineering, King Mongkut’s Institute of Technology Ladkrabang, Bangkok 10520, Thailand; 2 Department of Industrial Engineering, Faculty of Engineering, Rajamangala University of Technology Thanyaburi, Pathum Thani 12110, Thailand

**Keywords:** scaffold, freeze-drying technique, characterization, mixture design technique

## Abstract

Materials and manufacturing technologies are necessary for tissue engineering and developing temporary artificial extracellular matrices. In this study, scaffolds were fabricated from freshly synthesized titanate (Na_2_Ti_3_O_7_) and its precursor titanium dioxide and their properties were investigated. The scaffolds with improved properties were then mixed with gelatin to form a scaffold material using the freeze-drying technique. To determine the optimal composition for the compression test of the nanocomposite scaffold, a mixture design with three factors of gelatin, titanate, and deionized water was used. Then, the scaffold microstructures were examined by scanning electron microscopy (SEM) to determine the porosity of the nanocomposite scaffolds. The scaffolds were fabricated as a nanocomposite and determined their compressive modulus values. The results showed that the porosity of the gelatin/Na_2_Ti_3_O_7_ nanocomposite scaffolds ranged from 67% to 85%. When the mixing ratio was 100:0, the degree of swelling was 22.98%. The highest swelling ratio of 85.43% was obtained when the freeze-drying technique was applied to the mixture of gelatin and Na_2_Ti_3_O_7_ with a mixing ratio of 80:20. The specimens formed (gelatin:titanate = 80:20) exhibited a compressive modulus of 30.57 kPa. The sample with a composition of 15.10% gelatin, 2% Na_2_Ti_3_O_7_, and 82.9% DI water, processed by the mixture design technique, showed the highest yield of 30.57 kPa in the compression test.

## 1. Introduction

The scaffold is a structure required for cell growth. When living cells are in the scaffold, they multiply. Collagen, gelatin, silk, and synthetic PLA/PCL materials might be used for biomaterial scaffold development. In most cases, the biomaterial scaffold is used in experiments with various methods. [1] Currently, research is being conducted to develop biomaterials for medical applications. However, while developing material properties, the improvement of the mechanical properties of materials should be considered. The mechanical properties of materials can respond positively to real applications, especially the mechanical properties of gelatin. The mechanical properties of gelatin must be improved before it is used. [2] Gelatin consists of protein molecules composed of amino acid chains. Gelatin can be obtained from bones and animal skins. When gelatin is melted at 32 °C, it is in a viscous liquid state. Then, it becomes a gel within a few minutes. Gelatin is mainly used as an ingredient in cosmetics, medicines, foods, photographic films, and coated tablets. However, gelatin is too expensive for commercial use. Therefore, the properties of gelatin were investigated in several studies. The improvement of the properties of gelatin is usually performed by mixing it with other materials to reduce the material cost [2,3,4,5,6,7]. The use of titanium oxide (TiO_2_) in the culture of muscle cells (C2C12 myoblasts), fibroblasts, and keratinocytes has been extensively studied. These cultured cells showed good adhesive properties and could be efficiently used in tissue engineering [8,9]. Nano titanium dioxide is a biocompatible material with high tensile strength and nontoxic effects [10,11,12]. It can improve the antibacterial properties of the scaffold material and promote the wound-healing performance of the material [13,14,15]. Scaffolds have also been prepared by salt leaching [16,17,18], solvent casting [19], phase separation [20], gas foaming [21], freeze drying, electrospinning, and fiber bonding [22,23]. The gas foaming technique offers several advantages in the fabrication of scaffolds, such as no need for aggressive organic solvents during the fabrication process, and the pore size and fiber diameter can be adjusted. However, this technique also has some disadvantages, such as the absence of pore interconnections, which in turn depend on the expansion mechanism of the gas during scaffold formation. The electrospinning processes offer simple advantages such as cost efficiency, wide pore size distribution, high porosity, and large surface areas. Therefore, electrospinning processes are used to produce samples with small pores and inhomogeneous pore distribution. However, the process leads to the formation of scaffolds with a low volume. Highly porous samples with interconnected pores can be produced using the fiber-bonding technique. However, a major limitation of this technique is that the prepared samples are toxic to cells, and this toxicity cannot be eliminated [24]. Additionally, several devices, a large room, and numerous protective systems are required to perform the gas foaming, electrospinning, and fiber-bonding techniques. In comparison, the freeze-drying technique is a simple and inexpensive choice because only solvents are required in this method [25]. A mixture of titanium dioxide (TiO_2_) and polymers forms a framework that is more powerful due to an improvement in mechanical properties. A mixture of titanium dioxide (TiO_2_) and polymers results in better performance compared to the use of a normal polymer regarding size and texture [26,27,28]. Several studies have demonstrated the effectiveness of titanium dioxide (TiO_2_) in killing bacteria [29,30,31,32,33,34,35]. Some studies have shown that the use of nanoparticles along with a polymer improves the properties of the scaffold. The use of nanoparticles in a proportion of 5% mixed with 25–40% microcomposites can improve the properties [36,37,38,39]. The physical properties of titanium dioxide, such as structure, particle size, specific surface area, etc., directly affect the reaction mechanism. These physical properties depend on the synthesis method and conditions. In this study, the hygrothermal method was used to synthesize the material. In this method, the shape of the compounds can be controlled. Compounds with various shapes have been reported, including those with nanostructures such as nanotubes [40,41,42,43], nanoribbons [44,45,46,47], and nanowires [48,49]. The hydrothermal synthesis method increases chemical homogeneity without the need for further combustion or comminution. Additionally, the hydrothermal method allows the synthesis of compounds with a layered structure, especially a layered structure in which ions move between layers [40,49]. The hydrothermal synthesis method uses titanium dioxide (TiO_2_), and thus, the synthesis process must be performed under highly alkaline conditions and at high temperatures. However, such alkaline conditions and high temperatures can accelerate the process of structural changes in the compound. For example, one study reported that the layered structure of titanium dioxide (TiO_2_) changes to Na_2_Ti_3_O_7_ [50].

Some studies [51,52] reported the preparation of a gelatin/Na_2_Ti_3_O_7_ nanocomposite scaffold by mixing titanate (Na_2_Ti_3_O_7_) and gelatin. A salt leaching technique was used to synthesize titanate (Na_2_Ti_3_O_7_) from titanium dioxide (TiO_2_). The prepared gelatin/Na_2_Ti_3_O_7_ nanocomposite scaffold showed the best results concerning tensile strength and pore size. In this study, a mixture-design method was used to fabricate the scaffold instead of the freeze-drying technique. Then, the porosity, compression modulus, biodegradability, and swelling of the prepared samples were investigated.

## 2. Materials and Methods

### 2.1. Materials

Two main materials were used in the experiments. The first was gelatin which comprised porcine skin, 180 G Bloom, Type B from Fluke Analytical, and 2,2,2-trifluoroethanol (TFE) (purity 99.0%). Powdered gelatin was obtained from Sigma-Aldrich Corporation, Bangkok, Thailand.

The other material was titanate (Na_2_Ti_3_O_7_) synthesized from titanium dioxide and sodium hydroxide as precursors. A solution of sodium hydroxide (20 mL) was mixed with 0.5 g of titanium dioxide and then stirred with a magnetic stirrer (mixing time: 90 min). The mixture was then placed in an alkaline hydrothermal reactor at 200 °C for 24 h. The slurry was then purified with deionized water until the pH reached 7 and titanate (Na_2_Ti_3_O_7_) was formed. Then, the titanate was dried in an oven at 80 °C for 24 h to obtain titanate (v). The titanate had a specific surface area (BET) of 10 m^2^/g, an average primary particle size of 197 nm, and a catalytic activity equivalent to that of 100% anatase.

### 2.2. Mixture Design

The mixture design experiment involves analyzing the result as a function of the percentage of components [53]. The objective is to explore the possible results by estimating the parameters of each component to be prepared to achieve the best value or the value desired by the experimenter. In the present study, the constrained mixture design principle or the vertex model was used. The experimental design was based on three parameters: gelatin, Na_2_Ti_3_O_7_, and DI water. Minitab 21 software was used for statistical analyses. The software was customized to display the three factors as A, B, and C, where A was gelatin, B was Na_2_Ti_3_O_7_, and C was DI water. Gelatin and collagen were used for fabricating the scaffold for the fibroblast cell culture. The properties of the scaffolds obtained from type A and type B gelatin were compared to the scaffolds obtained from collagen, which is used widely as a skin substitute. Titanate ribbon (Na_2_Ti_3_O_7_) was synthesized to study the compressive modulus. Table 1 presents the ratio in which the three substances (A, B, and C) were mixed.

### 2.3. Preparation of the Gelatin/Na_2_Ti_3_O_7_ Nanocomposite Scaffold Samples Using the Freeze-Drying Technique

**Figure 1:**
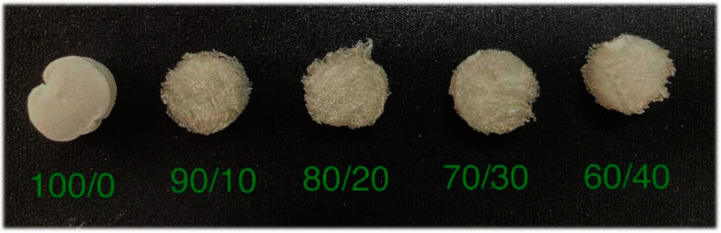
The gelatin/Na_2_Ti_3_O_7_ nanocomposite scaffold samples prepared using the freeze-drying technique.

According to the principles of mixture design, the three raw materials (gelatin, titanate, and DI water) were mixed in a definite ratio and stirred for 30 min at 50 °C. The mixtures were then poured into the wells of a five-well culture plate, with each well containing equal volumes, followed by freezing treatment overnight at −30 °C. After vacuum freeze-drying at −50 °C, all the samples were stored at −20 °C until further use [10]. The mass ratios and the designations of the prepared samples are listed in Table 2. Some of the mixtures are shown in Figure 1.

### 2.4. Compressive Test

**Figure 2:**
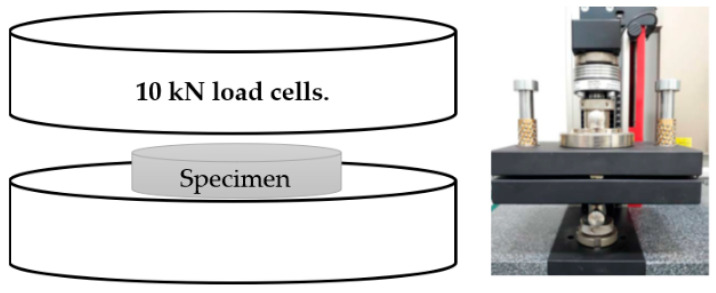
The testing of the prepared gelatin/Na_2_Ti_3_O_7_ nanocomposite scaffold samples using the universal testing machine.

Pressure resistance is one of the key properties of scaffolds. Therefore, the impact of the prepared gelatin/Na_2_Ti_3_O_7_ nanocomposite scaffolds was investigated by employing the universal testing machine (UTM, Instron No. 5566, Norwood, MA, USA) using the 10 kN load cells (Figure 2). The flexible cellular materials slab and the bonded and molded urethane foams were evaluated using the respective standard ASTM D 3574 methods [18]. The testing was conducted in a dry state at 25 °C at a speed maintained at 0.5 mm/min. The load was applied until the samples were compressed to approximately 100% of their original height. The stiffness of the scaffolds was assessed at the stress-point region. The yield strength was considered the stress-strain kPa yield point. The dimensions of the prepared samples were diameter, 1 cm and thickness, 1 cm. Each sample was tested five times. Subsequently, the average and standard deviations were determined for all samples.

### 2.5. Biodegradation of the Gelatin/Na_2_Ti_3_O_7_ Nanocomposite Scaffold Samples

The bioavailability of the prepared gelatin/Na_2_Ti_3_O_7_ nanocomposite scaffold samples was determined through the in vitro weight biodegradation analysis of the samples. The sample fragments were first incubated in the PBS solution (pH 7.4) containing 104 U/mL of lysozyme in an oven at 37 °C for 54 h. Afterwards, the samples were incubated in an enzyme-free buffer, followed by curing and then thoroughly washing the samples with pure water to remove the enzymes. The samples were then dried and weighed several times, i.e., the degree of enzymatic biodegradation was determined after 0.5, 1, 1.5, 24, 48, and 54 h [16,17] using the following equation:

(1) $$\mathrm{Weight remain}\left(\%\right)=100- [\frac{\left(W_{0}-W_{f}\right)}{W_{0}}\times 100]$$

where:

$W_{0}$ denotes the initial weight of the nanocomposite scaffold;
$W_{f}$ denotes the final weight of the nanocomposite scaffold.

### 2.6. Swelling Test

The swelling test of scaffolds is an important tool to investigate the diffusion of water or liquid into the scaffold. In the present study, the swelling test was performed to evaluate the percentage difference between the dry weight and wet weight of the prepared biomedical scaffold. The dried nanocomposite scaffold was weighed and then soaked in a PBS buffer solution (pH 7.4) at 37 °C for 3 h [20,21]. Afterwards, both sides of the scaffold were wiped using low-lint paper for 10 s on each side, followed by weighing the scaffold immediately. The dry weight and wet weight values were then utilized to calculate the swelling ratio using the following formula:

(2) $$\mathrm{Swelling ratio}=\frac{W_{so}-W_{o}}{W_{0}}$$

where:

$W_{so}$ denotes the weight of the scaffold absorbing the water content;
$W_{0}$ denotes the initial weight of the nanocomposite scaffold.

### 2.7. Statistical Analysis

The quantitative results were evaluated for data normality using Explore software and subjected to Tukey’s A test. The results were presented as the mean standard error (SE). In the case of significant result values obtained (*p* = 0.05), post hoc tests were conducted. Post hoc tests are an integral part of ANOVA. When ANOVA is performed to compare the means of a minimum of three groups, obtaining statistically significant results indicates that all the compared group means are not equal. However, the ANOVA results do not identify which particular differences between the pairs of means are significant. In this context, using post hoc tests enables exploring the differences among multiple groups while controlling the experiment-wise error rate. Tukey’s method is a single-step multiple-comparison procedure and statistical test, which may be used to identify the means that are significantly different from each other. Therefore, Tukey’s multiple-comparison test was also conducted for analysis in the present study. All descriptive and inferential statistical analyses were conducted using MINITAB Statistical Software (v. 21).

## 3. Results and Discussion

### 3.1. Experimental Design and Results

A simplex axial design experiment was conducted for the mixture design of gelatin (A), titanate (Na_2_Ti_3_O_7_) (B), and DI water (C) using the conditions stated above. In total, 27 experimental trials were performed, with three replicates in each trial condition. The results of the compressive test are presented in Table 2.

### 3.2. Residual Plots for Response

**Figure 3:**
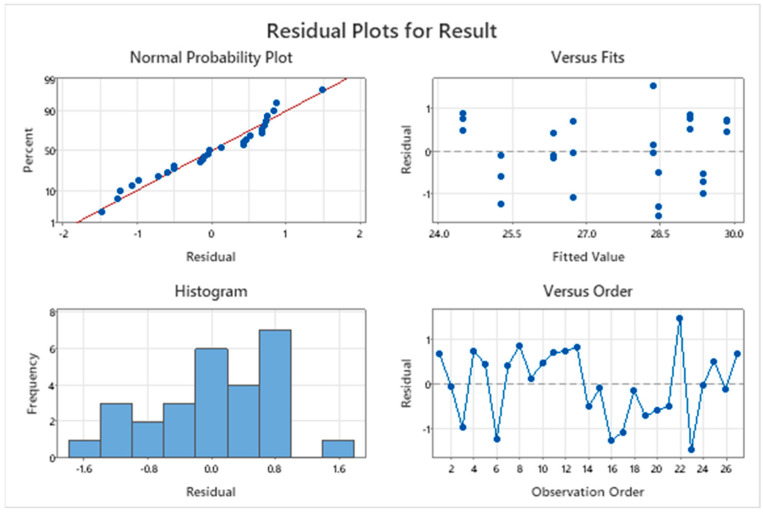
The residual plots for response average (kPa).

The quality of the result data was evaluated using the (1) normal distribution test, (2) the data independence test, and (3) the test of variance stability (Figure 3). As depicted in Figure 3, the data distribution did not have a specific pattern, indicating data independence, i.e., the data were collected randomly. In addition, the plot in which a straight line indicated the normality of the data obtained in the experimental work demonstrated that the experimental data had a stable variance as per the design. Therefore, it was concluded that the obtained data had all three properties.

### 3.3. Analysis of Variance (ANOVA)

The analysis of variance, or ANOVA, is a linear modeling method for evaluating the relationship data [16]. In the present study, an ANOVA was performed to determine the compressive modulus. The results were considered statistically significant at *p* < 0.05 (Table 3). The ANOVA results revealed that the interaction term between the amount of gelatin used and the amount of titanate (Na_2_Ti_3_O_7_) used was statistically significant (*p* = 0.000). The amounts of gelatin and DI water were also significantly related (*p* < 0.05). The coefficient of determination (R2) indicates the percentage variation in the dependent variables that is explained by the independent variables in a regression analysis. The results for the regression analysis conducted in the present study for the response surface methodology results presented in Table 3 revealed a high coefficient of determination (R2 = 84.55%). This observation indicated that the independent variables [the amounts of gelatin, titanate (Na_2_Ti_3_O_7_), and DI water] could explain 82.54% of the variation in the independent variables [the amounts of gelatin, titanate (Na_2_Ti_3_O_7_), and DI water]. Therefore, the model was used to obtain a prediction equation to determine an accurate and appropriate response value.

### 3.4. Results of the Response Surface Methodology Analysis

**Figure 4:**
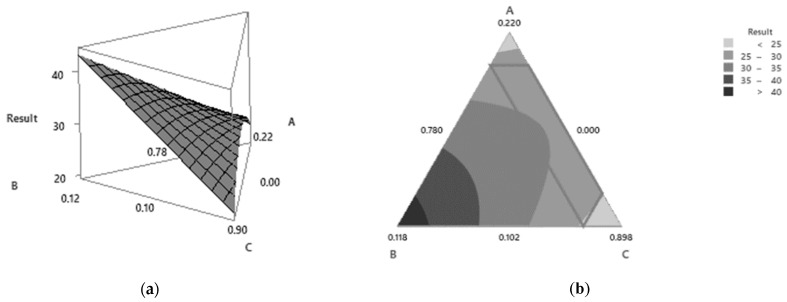
The mixture surface plot (**a**) and the mixture contour plot (**b**).

The response surface methodology analysis is an effective method for optimizing the process conditions. It may also be used for determining the influence of various factors. As depicted in Figure 4, the response surface revealed for the compressive test of the nanocomposite scaffold-forming process involving gelatin, titanate (Na_2_Ti_3_O_7_), and DI water increased with the increase in the amounts of gelatin and Na_2_Ti_3_O_7_.

### 3.5. Optimization

**Figure 5:**
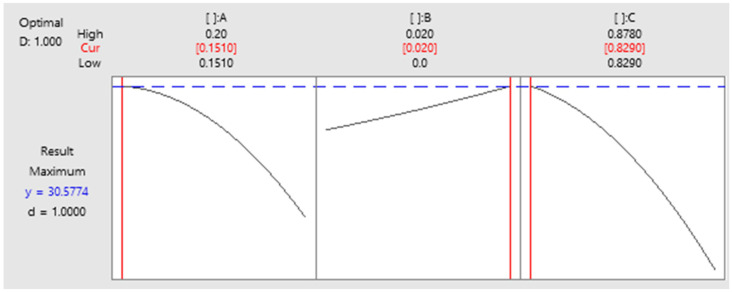
Optimization chart.

A response optimizer function was adopted to determine the most appropriate value of the factors and thereby efficiently obtain the tensile strength. In addition, another function was adopted to identify the most appropriate parameter among the factors and measure composite desirability (D). The value of composite desirability ranged from 0 to 1. When D was equal to 1, the result was favorable for the overall response. The results revealed that 15.10% gelatin, 2% titanate (Na_2_Ti_3_O_7_), and 82.90% DI water, or gelatin/Na_2_Ti_3_O_7_ in the ratio of 80/20, yielded the highest tensile strength of 30.57 kPa and the highest desirability of 1 (Figure 5).

The samples with gelatin and Na_2_Ti_3_O_7_ mixed in ratios of 100/0, 90/10, 80/20, 70/30, and 60/40 were investigated using the degradation test and the swelling test and also by analyzing the surface morphology and the pore size of the samples.

### 3.6. Biodegradation of the Gelatin/Na_2_Ti_3_O_7_ Nanocomposite Scaffold Samples Results

**Figure 6:**
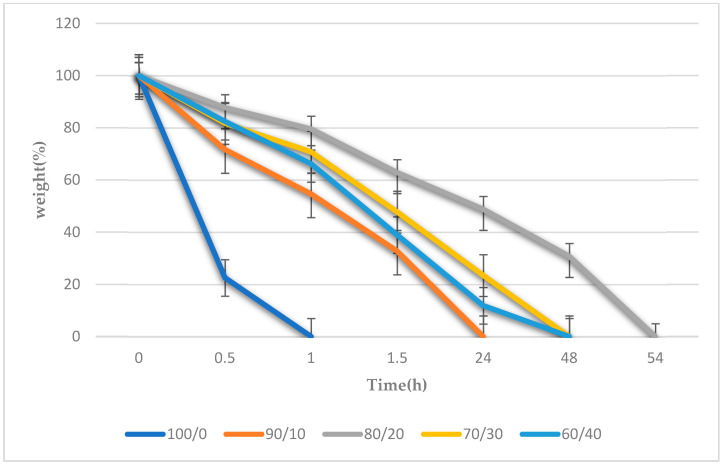
Results of the biodegradation of the gelatin/Na_2_Ti_3_O_7_ nanocomposite scaffolds. (n = 20) at *p* < 0.05.

Figure 6 depicts a comparison of the pure gelatin nanocomposite scaffolds and the gelatin/Na_2_Ti_3_O_7_ nanocomposite scaffolds based on the results of the biodegradation analysis [18,19]. It was observed that the nanocomposite scaffolds prepared from pure gelatin (gelatin/Na_2_Ti_3_O_7_ ratio—100/0) degraded in just one hour, while the nanocomposite scaffolds prepared from the 90/10 gelatin/Na_2_Ti_3_O_7_ mixture required 24 h for degradation. Moreover, the 60/40 and 70/30 gelatin/Na_2_Ti_3_O_7_ mixtures degraded in 48 h, while the 80:20 gelatin/Na_2_Ti_3_O_7_ mixture degraded in 54 h.

### 3.7. Swelling Test Results

**Figure 7:**
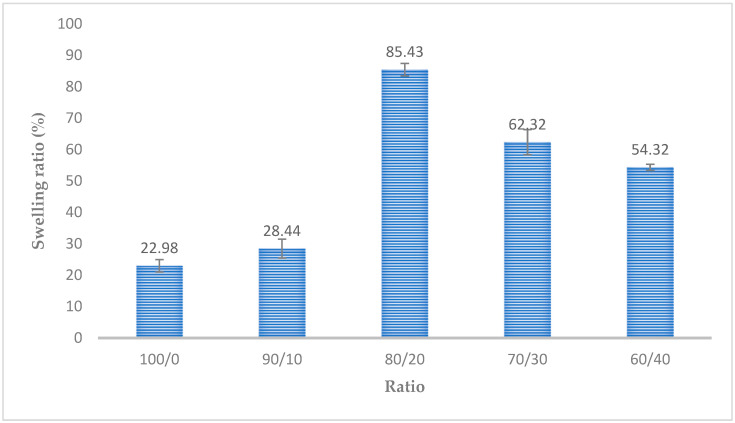
The swelling ratios of the different gelatin/Na_2_Ti_3_O_7_ nanocomposite scaffolds prepared using the freeze-drying technique. (n = 20) at *p* < 0.05.

Figure 7 depicts the nanocomposite scaffolds obtained using the freeze-drying technique. The swelling ratio of the scaffold with a gelatin/Na_2_Ti_3_O_7_ mixture ratio of 100:0 was 22.98%. The highest swelling ratio of 85.43% was obtained for the scaffold with a gelatin/Na_2_Ti_3_O_7_ mixture ratio of 80:20. This corresponded to the porosity value of the mixing ratio of 80:20, i.e., when the workpiece had high porosity, there was more space for water absorption, and thus, the rate of swelling increased.

### 3.8. Porosity Percent and Pore Size of the Gelatin/Na_2_Ti_3_O_7_ Nanocomposite Scaffolds Prepared Using the Freeze-Drying Technique

The porosity of the gelatin/Na_2_Ti_3_O_7_ nanocomposite scaffolds was determined using the following equation [25]:

(3) $$Porosity\left(\%\right)=\frac{V_{pore}}{V_{compact}}\times 100$$

where ***V_Pore_*** denotes the volume of the pores in the cellular scaffold material and ***V_compact_*** denotes the volume of the nonporous workpiece.

**Figure 8:**
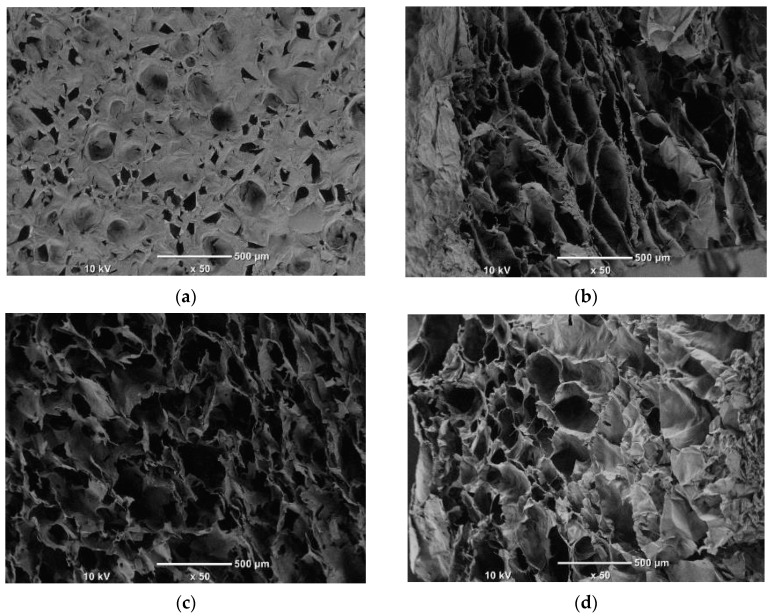

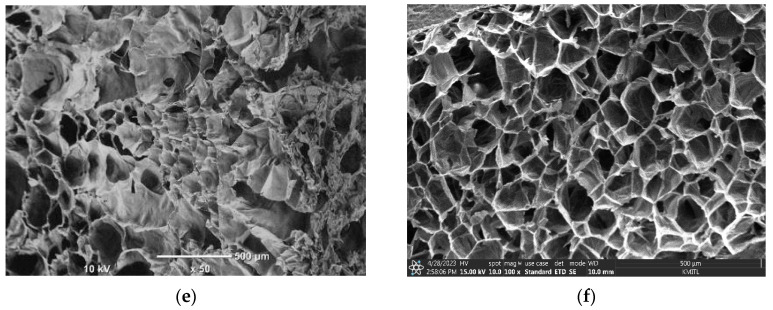
The SEM images of the scaffolds prepared using the freeze-drying technique, with the gelatin/Na_2_Ti_3_O_7_ mixture ratio of (**a**) 100/0, (**b**) 90/10, (**c**) 80/20, (**d**) 70/30, (**e**) 60/40 and cross-sectional (**f**) 80/20.

The porous nature of the character scaffolds prepared using the freeze-drying technique depends on the size of the pore networks that are interconnected. In the present study, the scaffolds presented a homogeneous porous morphology. The porosity of the gelatin/Na_2_Ti_3_O_7_ nanocomposite scaffold was subject to the conditions of the freeze-drying technique. The porosity was calculated using Equation (3). The result revealed that the porosity of the samples ranged from 67% to 85% (Figure 6). The porosity of the gelatin/Na_2_Ti_3_O_7_ nanocomposite scaffolds was >85%, which is conducive to cell growth [16,17,18]. The microstructures of the pure gelatin and gelatin/Na_2_Ti_3_O_7_ nanocomposite scaffolds are depicted in Figure 8a–e. The pure gelatin scaffolds were observed to be spongy, with interconnected pore networks of indeterminate sizes distributed throughout the scaffold. The gelatin/Na_2_Ti_3_O_7_ scaffolds had a porous material, with evenly-distributed pores of sizes in the approximate range of 250–425 µm, which is the optimal size for the growth of bone cells [54]. However, this variation in the pore size of the prepared scaffolds could affect the movement of toxins for removal from the cells and the transmission of nutrients through the scaffold material. Despite the afore-stated limitation of the prepared scaffold samples, the fact that all prepared samples exhibited a certain degree of porosity indicated that these scaffolds would influence cell growth upon application [16,17,18].

The porosity percentage and the pore diameter (pore size) of the gelatin/Na_2_Ti_3_O_7_ nanocomposite scaffolds prepared in the present study were determined, and the result values are listed in Table 4. As evident, the pore size decreased when excessive or pure Na_2_Ti_3_O_7_ was added to the material composition [51,52]. In addition, the diameters of the pores (pore size) in the different gelatin/Na_2_Ti_3_O_7_ scaffolds were not extremely small or large.

### 3.9. Surface Morphology of the Prepared Nanocomposite Scaffolds

The scanning electron microscope (SEM) [1,11] was invented by M. Von Andenne in 1957. In SEM analysis, the sample preparation step is crucial for obtaining the representative material and ensuring that the results truly refer to the structure. However, the samples are not required to be of the same size. In the present study, only the surfaces of the sample scaffold structures were examined using SEM. Therefore, the number of electrons passing through the scaffold to the transmission electron microscope was not determined. Scanning electron microscopy may, therefore, be performed for analyzing the surfaces of cells and skin samples. A high-energy electron beam was aimed at the surface of the scaffold sample. When the electron beam strikes the outer surface of the sample at higher angles of incidence, the scanning electron microscope produces secondary electrons. The signals from these secondary electrons are then recorded and converted to electronic signals. The electronic signals are then projected onto a television which produces a 3D image of the sample scaffold structures. A scanning electron microscope has a lower vision capability and a lower magnification compared to a transmission electron microscope. Therefore, a scanning electron microscope is suitable for the investigation, analysis, morphological structure study, and detailed examination of the sample scaffold surfaces, such as the surface of a cell, a skin sample, the external surface of a tissue, the external surface of a cell, a metal cross-section, and the cross-section of a nonmetal material, among others.

The morphology of the nanocomposite scaffold was examined using a scanning electron microscope (SEM; JSM-5610LV, JEOL) at a voltage of 20 kV. The sample surface was first coated with gold [55]. The sample was then examined under SEM, and the pore size was measured at least 20 times. The calculated average-diameter values are listed in Table 4. The images of the pores in the prepared nanocomposite scaffold samples were obtained after the dehydrothermal treatment (DHT) of the samples at 140 °C for 48 h (Figure 6). The diameters of the pores of the gelatin/Na_2_Ti_3_O_7_ nanocomposite scaffold were observed to have changed. The fabricated scaffold exhibited varied pore sizes (Figure 6). When a greater amount of Na_2_Ti_3_O_7_ was added to the scaffold composition, the sizes of the pores were reduced. Figure 8c depicts the high variation in the pore sizes. As depicted in Figure 8a–f, the diameters of the pores in the prepared gelatin/Na_2_Ti_3_O_7_ nanocomposite scaffolds ranged from 120 to 240 µm. However, the SEM cross-section of the 80/20 sample shown in Figure 8f also indicates uniform porosity characteristics. This indicates that when titanate (Na2Ti3O7) is added, samples can have more porosity. There is a network of interconnections between the pores compared to the 100/0 sample.

### 3.10. Characterization of the Gelatin/Na_2_Ti_3_O_7_ Nanocomposite Scaffolds

**Figure 9:**
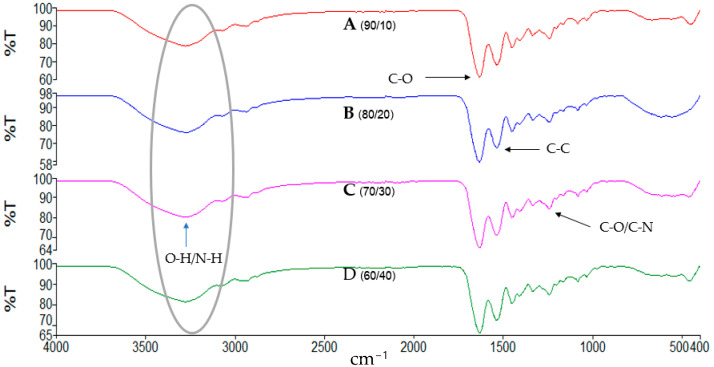
The FTIR spectrum of the gelatin/Na_2_Ti_3_O_7_ nanocomposite scaffold.

The FTIR spectra were obtained by analyzing the structure and molecular composition of the sample gelatin/Na_2_Ti_3_O_7_ nanocomposite scaffold using a Fourier transform infrared spectrometer (Brand: PerkinElmer Scientific Model: Spectrum Two FT-IR Spectrometer (Universal ATR Sampling Accessory)). By measuring the absorbance of substances in the infrared range (Figure 9), we found that in the wavelength range of 3300–3500 cm^−1^, aliphatic C–H stretching signals were found in the range of about 3100–2700 cm^−1^. Additionally, in the wavelength range of 1620–1680 cm^−1^, C = bonds were found. O stretching of amide groups was observed in the wavelength range of 1640–1590 cm^−1^; N–H bending signals were observed in the wavelength range of 1365–1455 cm^−1^; and C–H bending signals were observed in the wavelength range of 1256–1332 cm^−1^. Signs of C-N stretching were also found [56,57].

However, no new peaks were observed, indicating that the added titanate (Na_2_Ti_3_O_7_) did not react with glutaraldehyde. Titanate (Na_2_Ti_3_O_7_) can act as a reinforcing agent, dispersed in the hydrogel or matrix [58]. It can enhance the properties of the matrix and the composite materials.

### 3.11. Effects of the Different Techniques Used for the Fabrication of a Scaffold: Freeze-Drying Technique and Salt-Leaching Technique

The scaffolds prepared using the salt-leaching technique exhibit a porous nature that depends on the size of the salt crystals used. The porous networks in these scaffolds are interconnected, and the porous morphology is homogeneous. According to the results of the present study, the porosity of the prepared samples ranged from 75% to 81%. Previous studies [59,60,61] have not reported any scaffolds with a porosity exceeding 85%. Moreover, the porosity of the prepared scaffold was reported to be lower than that of the sample with added salt (at 85%) [62]. This resulted in the agglomeration of salt particles and a loss of salt particles during the sedimentation process. The results obtained in the present study using the freeze-drying technique for scaffold preparation were comparable to those of the previous studies [51]. The freeze-drying technique produced scaffolds with similar shapes and hole sizes. The pores were also well connected, while the porosity and the pore size could be controlled better using this technique.

## 4. Conclusions

The gelatin/Na_2_Ti_3_O_7_ nanocomposite scaffolds were fabricated using the freeze-drying technique, and then, they were characterized. The gelatin/Na_2_Ti_3_O_7_ nanocomposite scaffolds were prepared from gelatin and titanate (Na_2_Ti_3_O_7_). The mixture experimental-design technique was adopted as a statistical analysis tool. The results showed that titanate (Na_2_Ti_3_O_7_) played an important role in improving the porosity, compressive test results, biodegradation, swelling test outcomes, porosity percentage, and pore-size values of the prepared nanocomposite scaffolds. A good porosity range of 67–85% was observed. In the compressive test, the prepared samples (gelatin:titanate = 80:20) showed a yield value of 30.57 kPa. The sample prepared using the mixture design technique (15.10% gelatine, 2% Na_2_Ti_3_O_7_, and 82.9% DI water) resulted in the highest yield in the compressive test. Theoretically, a scaffold must be highly porous and have a three-dimensional shape. The pores should be interconnected to form networks throughout the scaffold to ensure adequate cell growth and the appropriate transport of nutrients and wastes. This is important because the transport of nutrients and wastes also controls the degradation rate and the water absorption capacity of the scaffold. Therefore, if the scaffold has a greater porous volume, cell-adhesion efficiency, and cell-growth volume are affected, which is consistent with the results of this study.

## Figures and Tables

**Table 1:** The raw materials used for synthesizing the gelatin/Na_2_Ti_3_O_7_ nanocomposite scaffolds.

Factors	Factor Levels	
	Low Level	High Level
A: Gelatin (g)	0	20
B: Na_2_Ti_3_O_7_ (g)	0	2
C: DI water (mL)	0	87.8

**Table 2:** The experimental design and results.

Run Order	Factors			Compressive Test (kPa.)
	A: Gelatin	B: Na_2_Ti_3_O_7_	C: DI Water	
01	0.200	0.000	0.800	27.407
02	0.200	0.002	0.798	26.764
03	0.122	0.000	0.878	24.678
04	0.120	0.002	0.878	25.240
05	0.161	0.001	0.839	30.306
06	0.180	0.001	0.819	29.861
07	0.180	0.002	0.818	28.396
08	0.141	0.001	0.858	28.511
09	0.140	0.002	0.858	27.186
10	0.200	0.000	0.800	26.687
11	0.200	0.002	0.798	26.222
12	0.122	0.000	0.878	24.038
13	0.120	0.002	0.878	25.370
14	0.161	0.001	0.839	30.586
15	0.180	0.001	0.819	29.621
16	0.180	0.002	0.818	28.866
17	0.141	0.001	0.858	28.337
18	0.140	0.002	0.858	26.974
19	0.200	0.000	0.800	25.647
20	0.200	0.002	0.798	26.182
21	0.122	0.000	0.878	25.170
22	0.120	0.002	0.878	24.970
23	0.161	0.001	0.839	30.546
24	0.180	0.001	0.819	29.941
25	0.180	0.002	0.818	28.659
26	0.141	0.001	0.858	29.871
27	0.140	0.002	0.858	27.953

**Table 3:** Summary of the results obtained in the analysis of variance (ANOVA).

Source	DF	Seq SS	Adj SS	Adj MS	F-Value	*p*-Value
Regression	3	87.933	87.933	29.3110	41.96	0.000
Linear	2	10.864	75.028	37.5141	53.70	0.000
Quadratic	1	77.069	77.069	77.0688	110.32	0.000
A*C	1	77.069	77.069	77.0688	110.32	0.000
Residual Error	23	16.067	16.067	0.6986		
Lack of Fit	5	11.408	11.408	2.2817	8.82	0.000
Pure Error	18	4.659	4.659	0.2588		
Total	26	104.000				
**S**	**R-sq**			**R-sq(adj)**	**PRESS**	**R-sq(pred)**
0.835807	84.55%			82.54%	21.6100	79.22%

**Table 4:** The porosity percentage and pore size of the scaffolds (significance level—*p* < 0.05).

Gelatin/Na_2_Ti_3_O_7_	Pore Size (µm)	Porosity (%)
100/0	124.23 ± 2.65	67.96 ± 2.77
90/10	195.32 ± 2.52	83.43 ± 0.82
80/20	278.71 ± 2.52	86.42 ± 1.08
70/30	257.79 ± 2.08	85.44 ± 1.20
60/40	234.56 ± 3.51	84.63 ± 2.30

## Data Availability

The data presented in this study are available in this article.
